# Association between systemic immune-inflammation index and atopic dermatitis: a cross-sectional study of NHANES 2001–2006

**DOI:** 10.3389/fmed.2024.1461596

**Published:** 2024-08-29

**Authors:** Qike Ding, Lihong Lin, Xiaoting Li, Xiaoping Xie, Tao Lu

**Affiliations:** Department of Dermatology, The First Affiliated Hospital of Shantou University Medical College, Shantou, Guangdong, China

**Keywords:** atopic dermatitis, systemic immune-inflammation index, cross-sectional study, National Health and Nutrition Examination Survey, outpatient US adults

## Abstract

**Background:**

While several studies have noted a higher SII correlates with multiple diseases, research on the association between SII and atopic dermatitis remains limited. Our cross-sectional study seeks to examine the association between SII and atopic dermatitis among outpatient US adults.

**Methods:**

This compensatory cross-sectional study utilized NHANES data from 2001–2006 cycles, conducting sample-weighted multivariate logistic regression and stratified analysis of sub-groups.

**Results:**

Higher levels of SII were positively associated with an increased risk of atopic dermatitis in adults with BMI <30 (OR, 1.44; 95% CI, 1.10–1.90) (*p* = 0.010).

**Conclusion:**

Our findings suggested SII higher than 330 × 10^9^/L was positively associated with a high risk of atopic dermatitis in US adults with BMI <30. To our knowledge, this is the first study focused on the risk of higher SII on atopic dermatitis in the outpatient US population. Currently, there are differences in the standards used to diagnose atopic dermatitis across countries, and our study may have implications.

## 1 Introduction

Atopic dermatitis is a common and chronic inflammatory dermatological condition that affects more than 200 million people worldwide, including 15 to 20% of children and 10% of adults (1, 2). It leads to substantial costs and accounts for the largest global burden of disability owing to skin diseases (3). Currently, there are many effective medications for the treatment of atopic dermatitis, such as JAK1 inhibitors and dupilumab. The mechanisms of action of these drugs involve the inhibition of inflammatory substances, which significantly control the condition of atopic dermatitis (4, 5). This represents a milestone compared to the effects achieved by traditional treatments.

Within the systemic inflammatory system of the human body, immune cells play a critical role in multiple diseases. Scientists have found that combined counts of lymphocytes, neutrophils, and platelets in the peripheral blood may be a better predictor of inflammatory status. Importantly, SII was calculated by counting three types of circulating immune cells: neutrophils, lymphocytes, and platelets. Moreover, SII was initially identified as a prognosis of kidney stones, hepatic steatosis, and others (6, 7). Atopic dermatitis has been reported to be associated with inflammatory responses (8–10). However, the impact of SII on atopic dermatitis in the outpatient US population is not fully elucidated, and little is known about its prognostic ability for atopic dermatitis. In this cross-sectional study using NHANES data, we tried our best to find the association between SII and atopic dermatitis among outpatient US adults (Table 1).

**Table 1:** Characteristics of participants in the NHANES 2001–2006 cycles.

Characteristic	Participants^a^	SII (10^9^/L)		*p* value
	Total (*N* = 9,097)	< 330 (*N* = 1,504)	≥ 330 (*N* = 7,593)	
Age (median [IQR])	40.00 [30.00, 48.00]	40.00 [30.00, 50.00]	40.00 [30.00, 48.00]	0.385
vitD (median [IQR])	60.60 [46.80, 75.40]	58.10 [43.40, 73.80]	61.10 [47.10, 75.40]	0.001
BMI (median [IQR])	27.10 [23.62, 31.54]	26.22 [23.10, 30.47]	27.24 [23.71, 31.75]	<0.001
Sex				<0.001
Male	4,267 (49.1)	897 (58.3)	3,370 (47.5)	
Female	4,830 (50.9)	607 (41.7)	4,223 (52.5)	
Marital status				0.595
Married	5,032 (58.0)	798 (57.2)	4,234 (58.2)	
Not Married	4,065 (42.0)	706 (42.8)	3,359 (41.8)	
Educational level				0.315
High school or less	4,386 (40.7)	715 (40.2)	3,671 (40.8)	
Some college	2,787 (33.0)	486 (34.9)	2,301 (32.7)	
College graduate or higher	1918 (26.3)	303 (24.9)	1,615 (26.6)	
Race and ethnicity^b^				<0.001
Mexican American	2009 (8.8)	269 (8.6)	1740 (8.9)	
Non-Hispanic White	4,363 (69.6)	524 (58.9)	3,839 (71.5)	
Non-Hispanic Black	1950 (11.4)	612 (23.5)	1,338 (9.3)	
Other	775 (10.1)	99 (9.0)	676 (10.3)	
Family income^c^				0.231
Low	2,365 (19.6)	379 (19.6)	1986 (19.6)	
Medium	3,170 (34.7)	542 (36.9)	2,628 (34.3)	
High	3,125 (45.8)	511 (43.5)	2,614 (46.2)	
Asthma^d^				0.16
No	7,945 (86.5)	1,334 (88.1)	6,611 (86.2)	
Yes	1,152 (13.5)	170 (11.9)	982 (13.8)	
Smoking status^e^				0.515
Never	4,917 (51.6)	800 (50.7)	4,117 (51.8)	
Former	1700 (20.2)	282 (21.7)	1,418 (19.9)	
Current	2,474 (28.2)	420 (27.6)	2054 (28.3)	
Atopic dermatitis				0.051
Yes	991 (12.4)	139 (10.4)	852 (12.8)	
No	8,106 (87.6)	1,365 (89.6)	6,741 (87.2)	

^a^Data are presented as unweighted number (weighted percentage) unless otherwise indicated. ^b^Race and ethnicity were self-reported. ^c^Categorized into the following 3 levels based on the family poverty income ratio: low income (≤1.3), medium income (>1.3 to 3.5), and high income (>3.5). ^d^Determined by answering the following question: “Has a doctor or other health professional ever told you that you have asthma?” ^e^We categorized smoking status into the following 3 groups: never smoked (smoked < 100 cigarettes in life), former smoker (smoked at least 100 cigarettes in life but has quit), and current smoker (smoked at least 100 cigarettes in life and is now still smoking).

## 2 Methods

### 2.1 Data sources

**Figure 1:**
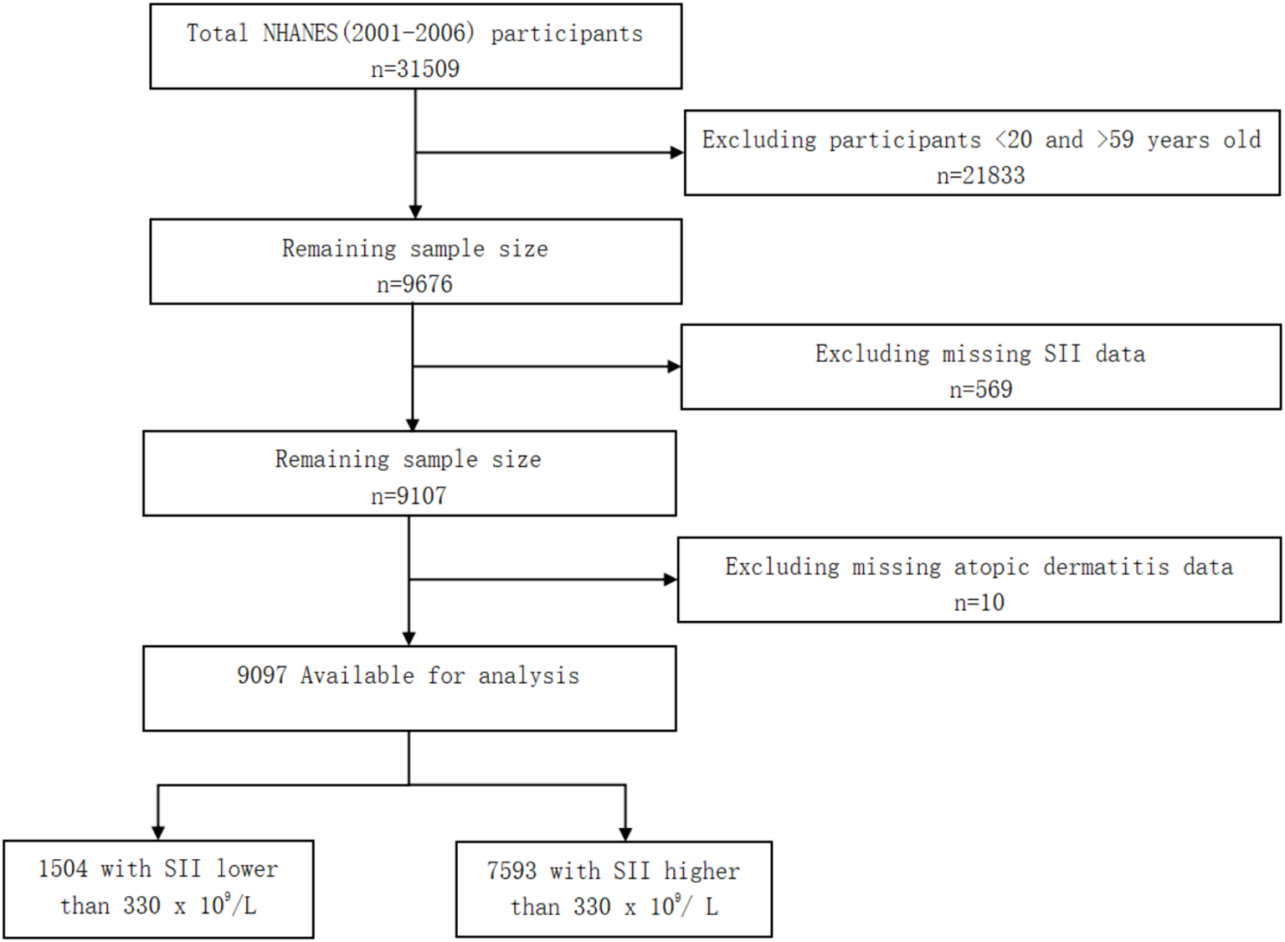
Flow diagram of the screening and enrollment of study participants.

Information on atopic dermatitis was only provided in the NHANES 2001–2006 cycles for adults 20 to 59 years of age. In this cross-sectional study, deidentified data for participants 20 to 59 years of age were extracted from the 2001–2006 NHANES cycles. This cross-sectional study was deemed exempt as it used publicly available data, and informed consent was waived. This study followed the STROBE reporting guideline. Data were analyzed from July to November 2023 (Figure 1).

### 2.2 Study design and population

Because information on atopic dermatitis was only provided in the NHANES 2001–2006 cycles for adults 20 to 59 years of age, we selected a total of 9,676 adults within this age range. Moreover, we excluded 569 participants who had missing SII data, 10 participants who had missing atopic dermatitis data. Finally, a total of 9,097 participants were involved. Of the 9,097 participants, there were 1,504 participants with SII lower than 330 × 10^9^/L and 7,593 participants with SII higher than 330 × 10^9^/L.

### 2.3 Assessment of SII and atopic dermatitis

SII was defined as (platelet x neutrophil)/lymphocyte (11). Throughout the analysis, we set the cutoff value at 330 × 10^9^/L based on previous studies of the NHANES (12, 13).

Atopic dermatitis was assessed by the question “Has a doctor ever told you that you have eczema?” (NHANES 2001–2004 cycles, Variable Name: DED061 and NHANES 2005–2006 cycle, Variable Name: AGQ180) (14) We refer to this inclusion criteria based on an article titled ‘Association between atopic dermatitis and thyroid disease among US adults in the 2001–2006 NHANES’ published in JAAD.

### 2.4 Covariates

The following covariates were included: sociodemographic variables (age, sex, race and ethnicity, family income, educational level, and marital status), NHANES cycles, BMI, serum 25(OH)D, asthma, and smoking status (15, 16). Races and ethnicities was categorized into 4 groups. Based on the family poverty income ratio, family income was categorized into the following 3 levels: low income (≤1.3), medium income (>1.3 to 3.5), and high income (>3.5). Educational level was categorized into 3 levels. Marital status was categorized into the following 2 groups: married, not married. We categorized smoking status into the following 3 groups: smoked <100 cigarettes in life, smoked at least 100 cigarettes in life but has quit now, and smoked at least 100 cigarettes in life and is now still smoking (17).

### 2.5 Statistical analysis

In accordance with NHANES analytic guidelines, our analyses considered the complex sampling design and sampling weights (18). The sampling weight was calculated using the following formula: fasting subsample 6-year mobile examination center (MEC) weight = fasting subsample 2-year MEC weight/3. Moreover, we used quartiles to describe the continuous variables with non-normal distribution and described the categorical variables using unweighted frequency and weighted percentage in this cross-sectional study.

We checked for multicollinearity using the VIF method. If the VIF was 5 or higher, it meant there was multicollinearity present (19). Model 1 was adjusted for sociodemographic variables and NHANES cycles. Model 2 was adjusted for sociodemographic variables, NHANES cycles, BMI (only for overall), serum 25(OH)D, asthma, and smoking status. We conducted a subgroup analysis on BMI to assess the possible influence of BMI on the association between SII and atopic dermatitis.

We used R version 4.3.1 for all the statistical analyses and we considered a significance level of *p* < 0.05 to show that the results were statistically significant.

## 3 Results

There were 31,509 participants from 2001–2006 included in this study. 21,833 participants <20 and > 59 years old were excluded, and 569 participants were excluded for missing SII data. After removing 10 participants missing atopic dermatitis data, 9,097 participants were finally enrolled. Compare with SII lower than 330 × 10^9^/L, participants with SII higher than 330 × 10^9^/ L were more non-Hispanic White (*p* < 0.001).

As shown in Table 2, logistic regression analysis found no significant difference between SII and atopic dermatitis after adjusting for covariates in the whole population. In the subgroup with BMI <30, the univariate and multivariate analyses demonstrated high SII over 330 × 10^9^/L was associated with a higher risk of atopic dermatitis in the crude model (OR, 1.53; 95% CI, 1.17–1.99; *p* = 0.002) and model 1 (OR, 1.47; 95% CI, 1.12–1.93; *p* = 0.006). After adjusting for all confounding factors, high SII over 330 × 10^9^/L was still positively associated with atopic dermatitis in the population with BMI <30 (OR, 1.44; 95% CI, 1.10–1.90; *p* = 0.010). The difference was not found in participants with BMI ≥30. Stratified logistic regression analysis in the subgroup with BMI <30 suggested no potential modifiers in the relationship between SII and atopic dermatitis in the population with BMI <30.

**Table 2:** Univariate and multivariate analyses by the activity-stratified logistic regression model, weighted.

BMI stratification	SII (10^**9**^/L)		*p* value
	< 330 (OR, 95% CI)	≥ 330 (OR, 95% CI)	
Overall			
Crude model^a^	1.0 (Reference)	1.26 (1.00–1.60)	0.051
Model 1^b^	1.0 (Reference)	1.18 (0.92–1.52)	0.178
Model 2^c^	1.0 (Reference)	1.15 (0.89–1.49)	0.272
< 30			
Crude model	1.0 (Reference)	1.53 (1.17–1.99)	0.002
Model 1	1.0 (Reference)	1.47 (1.12–1.93)	0.006
Model 2	1.0 (Reference)	1.44 (1.10–1.90)	0.010
≥ 30			
Crude model	1.0 (Reference)	0.85 (0.62–1.17)	0.474
Model 1	1.0 (Reference)	0.75 (0.53–1.06)	0.182
Model 2	1.0 (Reference)	0.73 (0.51–1.05)	0.084

BMI, body mass index; CI, confidence interval; OR, odds ratio; SII, systemic immune-inflammatory index. ^a^Crude model: adjusted for none. ^b^Adjusted for sociodemographic variables (age, sex, race and ethnicity, family income, educational level, and marital status) and NHANES cycles. ^c^Adjusted for sociodemographic variables, NHANES cycles, BMI (only for overall), serum 25(OH)D, asthma, and smoking status.

Because the percentage of missing data was small (missing rate varied from 0 to 4.8%) for any variable, no imputation method was used.

## 4 Discussion

Chronic inflammation is important in the development of atopic dermatitis (20, 21). SII was calculated by neutrophils, lymphocytes, and platelets (22). It reflects the inflammatory reactions and may be a useful diagnostic biomarker for systemic inflammatory activity. In this cross-sectional study, we identified higher SII level was independently associated with atopic dermatitis in participants with BMI <30. But, we did not find such an association in adults with BMI ≥30. Moreover, SII is a widely available method with a non-intrusive methodology, low cost, and easy accessibility. Therefore, SII can be identified as a biomarker for atopic dermatitis in participants with BMI <30. Currently, there are differences in the standards used to diagnose atopic dermatitis across countries, and our study may have implications.

Interestingly, our findings only demonstrated the association between SII and atopic dermatitis in participants with BMI <30. Because there are more risk factors in participants with BMI ≥30, the impact of SII on atopic dermatitis in participants with BMI ≥30 might be compensated by other confounding factors.

## 5 Conclusion

After adjusting for all confounding factors, high SII over 330 × 10^9^/L was still positively associated with atopic dermatitis in the population with BMI <30. From the above results obtained through the rigorous analysis, we believe that incorporating SII into the diagnostic criteria of atopic dermatitis may improve the diagnostic rate of atopic dermatitis. Early clinical manifestations of atopic dermatitis are often subtle and can be easily overlooked during diagnosis. Therefore, including SII as part of the diagnostic criteria may facilitate earlier detection and enable preventive management of the condition. Currently, there are differences in the standards used to diagnose atopic dermatitis across countries, and our study may have implications.

## Data Availability

Publicly available datasets were analyzed in this study. This data can be found at: https://www.cdc.gov/nchs/nhanes/index.htm.
